# Supplementary material for: Cryo-electron tomography reveals how COPII assembles on cargo-containing membranes
Source: Nat Struct Mol Biol. 2024 Nov 7;32(3):513–9. doi: 10.1038/s41594-024-01413-4 (PMC11919764; doi:10.1038/s41594-024-01413-4)
Supplement: Supplementary file 1 — Reporting Summary [file 41594_2024_1413_MOESM1_ESM.pdf]

nature portfolio

Corresponding author(s): Zanetti Giulia

Last updated by author(s): Aug 16, 2024

Reporting Summary

Nature Portfolio wishes to improve the reproducibility of the work that we publish. This form provides structure for consistency and transparency in reporting. For further information on Nature Portfolio policies, see our Editorial Policies and the Editorial Policy Checklist.

Statistics

For all statistical analyses, confirm that the following items are present in the figure legend, table legend, main text, or Methods section.

n/a	Confirmed	
☐	☒	The exact sample size (*n*) for each experimental group/condition, given as a discrete number and unit of measurement
☐	☒	A statement on whether measurements were taken from distinct samples or whether the same sample was measured repeatedly
☐	☒	The statistical test(s) used AND whether they are one- or two-sided<br>*Only common tests should be described solely by name; describe more complex techniques in the Methods section.*
☐	☒	A description of all covariates tested
☒	☐	A description of any assumptions or corrections, such as tests of normality and adjustment for multiple comparisons
☐	☒	A full description of the statistical parameters including central tendency (e.g. means) or other basic estimates (e.g. regression coefficient) AND variation (e.g. standard deviation) or associated estimates of uncertainty (e.g. confidence intervals)
☐	☒	For null hypothesis testing, the test statistic (e.g. *F*, *t*, *r*) with confidence intervals, effect sizes, degrees of freedom and *P* value noted<br>*Give P values as exact values whenever suitable.*
☒	☐	For Bayesian analysis, information on the choice of priors and Markov chain Monte Carlo settings
☒	☐	For hierarchical and complex designs, identification of the appropriate level for tests and full reporting of outcomes
☒	☐	Estimates of effect sizes (e.g. Cohen's *d*, Pearson's *r*), indicating how they were calculated

Our web collection on statistics for biologists contains articles on many of the points above.

Software and code

Policy information about availability of computer code

Data collection	SerialEM 4.1 beta, with PaceTOMO.
Data analysis	IMOD v4.11.15, Isonet v0.1, Dynamo v1.1.509, Relion v4.0, Relion v5.0 (4.1-alpha-1-commit-d2053c), https://github.com/EuanPyle/Membrane_Associated_Picking, MATLAB 2023a, Chimera, ChimeraX

For manuscripts utilizing custom algorithms or software that are central to the research but not yet described in published literature, software must be made available to editors and reviewers. We strongly encourage code deposition in a community repository (e.g. GitHub). See the Nature Portfolio guidelines for submitting code & software for further information.

Data

Policy information about availability of data

All manuscripts must include a data availability statement. This statement should provide the following information, where applicable:

- Accession codes, unique identifiers, or web links for publicly available datasets
- A description of any restrictions on data availability
- For clinical datasets or third party data, please ensure that the statement adheres to our policy

COPII Inner Coat on Vesicles: EMDB-19417

COPII Outer Coat (Rod) on Vesicles: EMD-19421

COPII Outer Coat (Vertex) on Vesicles: EMD-19418

COPII Inner Coat on Tubes with Sed5: EMDB-19410

COPII Outer Coat (Rod) on Tubes with Sed5: EMDB-19414

COPII Outer Coat (Vertex) on Tubes with Sed5: EMDB-19416
COPII Outer Coat (5-way vertex) on vesicles: EMDB-19879

Research involving human participants, their data, or biological material

Policy information about studies with human participants or human data. See also policy information about sex, gender (identity/presentation), and sexual orientation and race, ethnicity and racism.

Reporting on sex and gender	N/A
Reporting on race, ethnicity, or other socially relevant groupings	N/A
Population characteristics	N/A
Recruitment	N/A
Ethics oversight	N/A

Note that full information on the approval of the study protocol must also be provided in the manuscript.

Field-specific reporting

Please select the one below that is the best fit for your research. If you are not sure, read the appropriate sections before making your selection.

☒ Life sciences ☐ Behavioural & social sciences ☐ Ecological, evolutionary & environmental sciences

For a reference copy of the document with all sections, see nature.com/documents/nr-reporting-summary-flat.pdf

Life sciences study design

All studies must disclose on these points even when the disclosure is negative.

Sample size	Total numbers of contributing particles for subtomogram averaging are stated in Materials and methods. The sample size was determined by the availability of data collection time.
Data exclusions	Tilt series were discarded on occasions where accurate tomogram reconstruction could not be achieved. Tilt images were excluded if mistracked or if contaminating objects obscured the view.
Replication	For subtomogram averaging, all datasets were divided into two halves for independent processing . Map resolution was determined by half-map Fourier Shell Correlation consistent with gold standard methods.
Randomization	Particles falling into the two halves for independent processing were selected randomly by RELION5 intrinsic routines
Blinding	Not relevant, as data processing is done following automatic procedures

Reporting for specific materials, systems and methods

We require information from authors about some types of materials, experimental systems and methods used in many studies. Here, indicate whether each material, system or method listed is relevant to your study. If you are not sure if a list item applies to your research, read the appropriate section before selecting a response.

Materials & experimental systems

n/a	Involved in the study	
☒	☐	Antibodies
☐	☒	Eukaryotic cell lines
☒	☐	Palaeontology and archaeology
☒	☐	Animals and other organisms
☒	☐	Clinical data
☒	☐	Dual use research of concern
☒	☐	Plants

Methods

n/a	Involved in the study	
☒	☐	ChIP-seq
☒	☐	Flow cytometry
☒	☐	MRI-based neuroimaging

Eukaryotic cell lines

Policy information about cell lines and Sex and Gender in Research

Cell line source(s)	Sf9 insect cells for expression are from invitrogen
Authentication	No authentication done
Mycoplasma contamination	Not assessed
Commonly misidentified lines (See ICLAC register)	No commonly misidentified cell lines were used

Plants

Seed stocks	N/A
Novel plant genotypes	N/A
Authentication	N/A
