# Supplementary figures and images for: Cryo-electron tomography reveals how COPII assembles on cargo-containing membranes
Source: Nat Struct Mol Biol. 2024 Nov 7;32(3):513–9. doi: 10.1038/s41594-024-01413-4 (PMC11919764; doi:10.1038/s41594-024-01413-4)

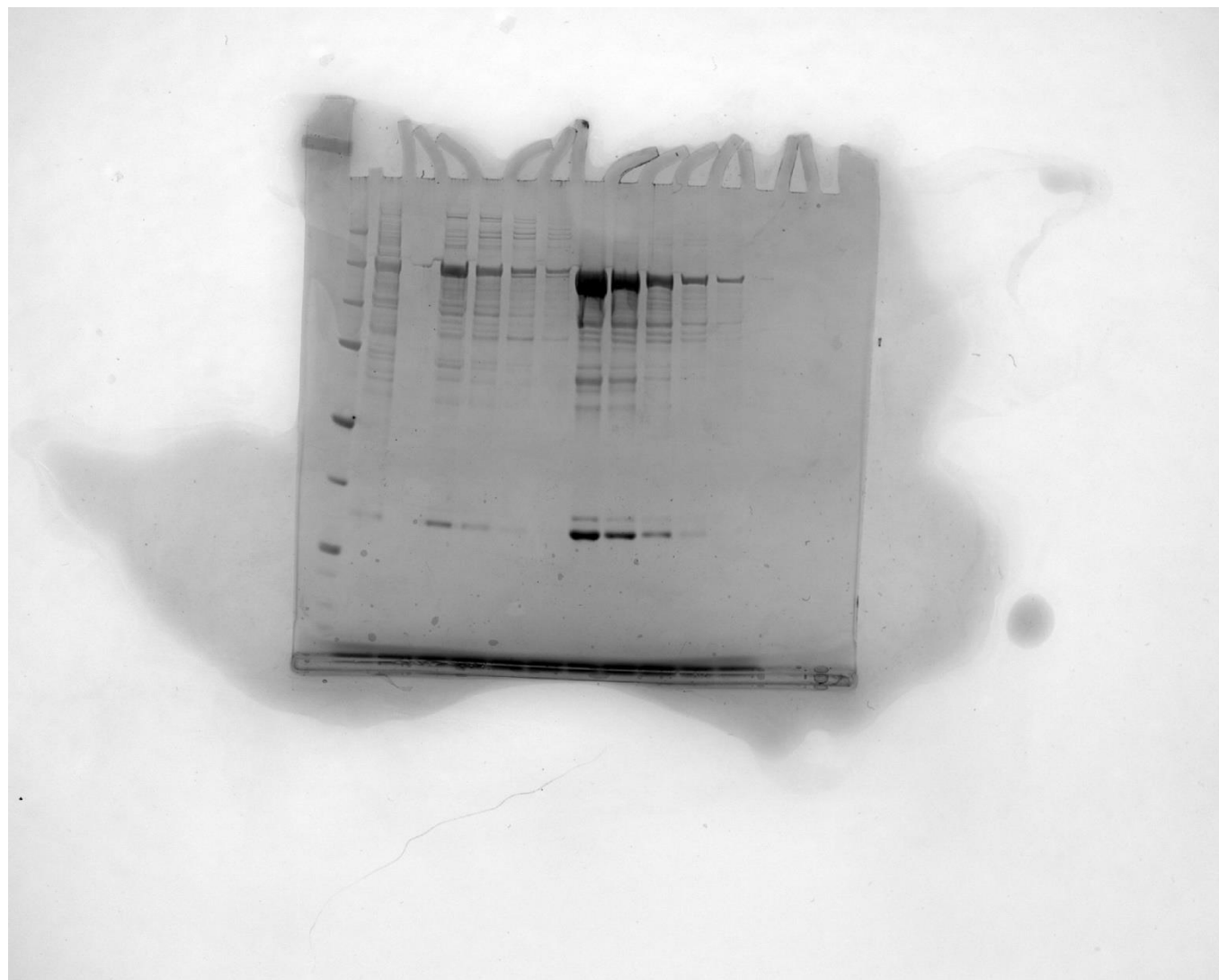

Sec13-Sec31 purification gel (Extended data Fig 1A)

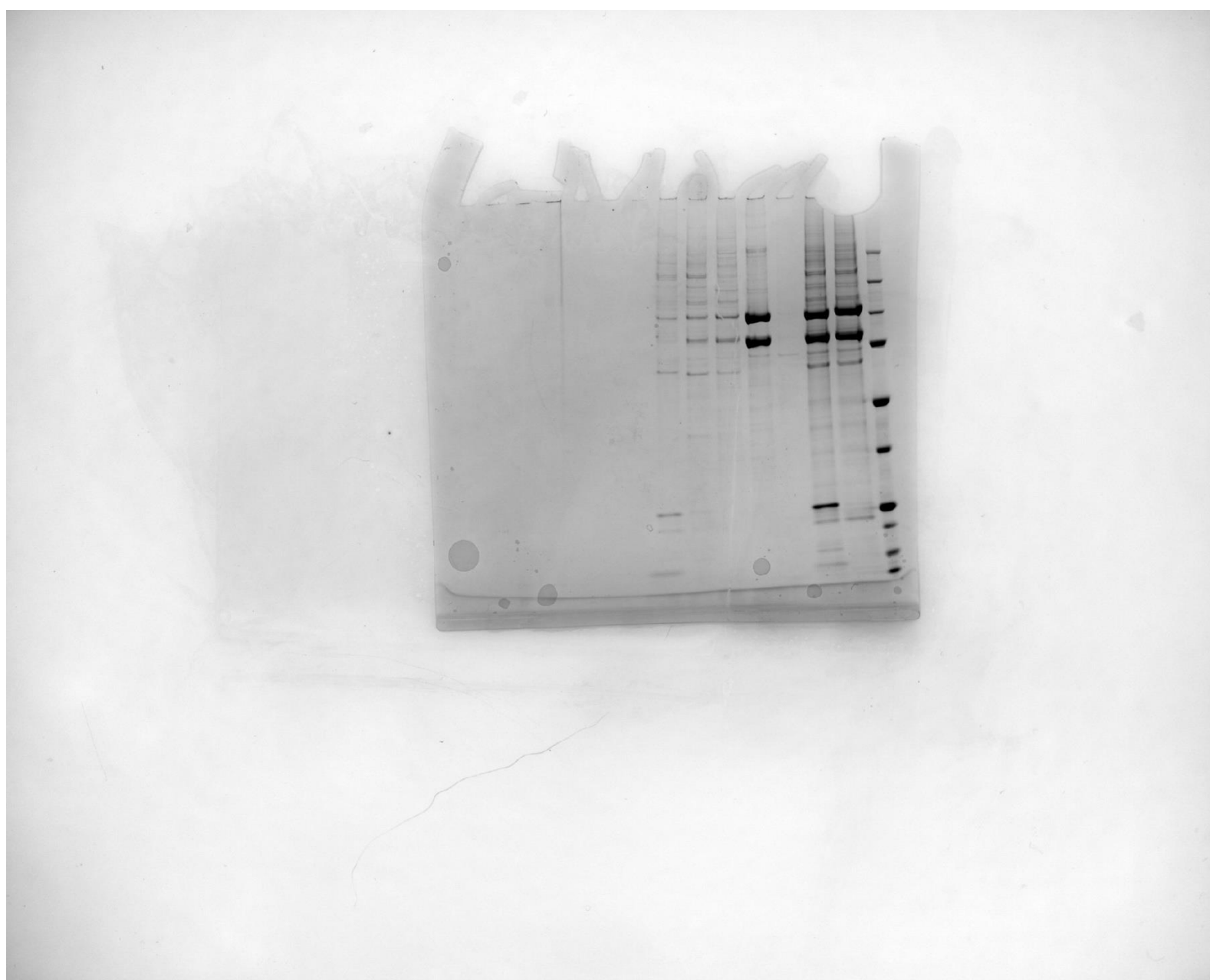

Sec23-Sec24 purification gel (Extended data Fig 1A)

Supplement: Supplementary file 4 — Unprocessed gels. [file 41594_2024_1413_MOESM4_ESM.pdf]

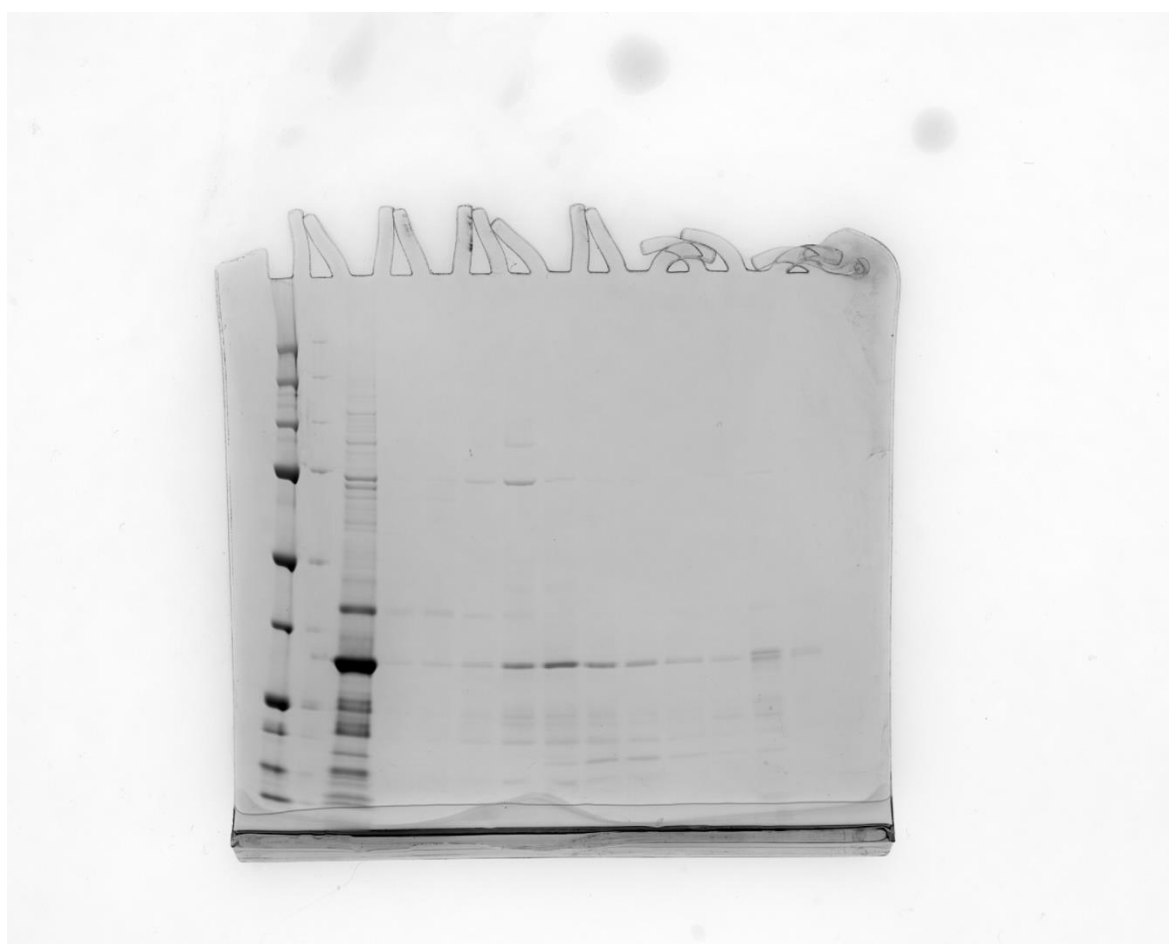

Sed5\_purification\_gel (Extended data Fig 2B)

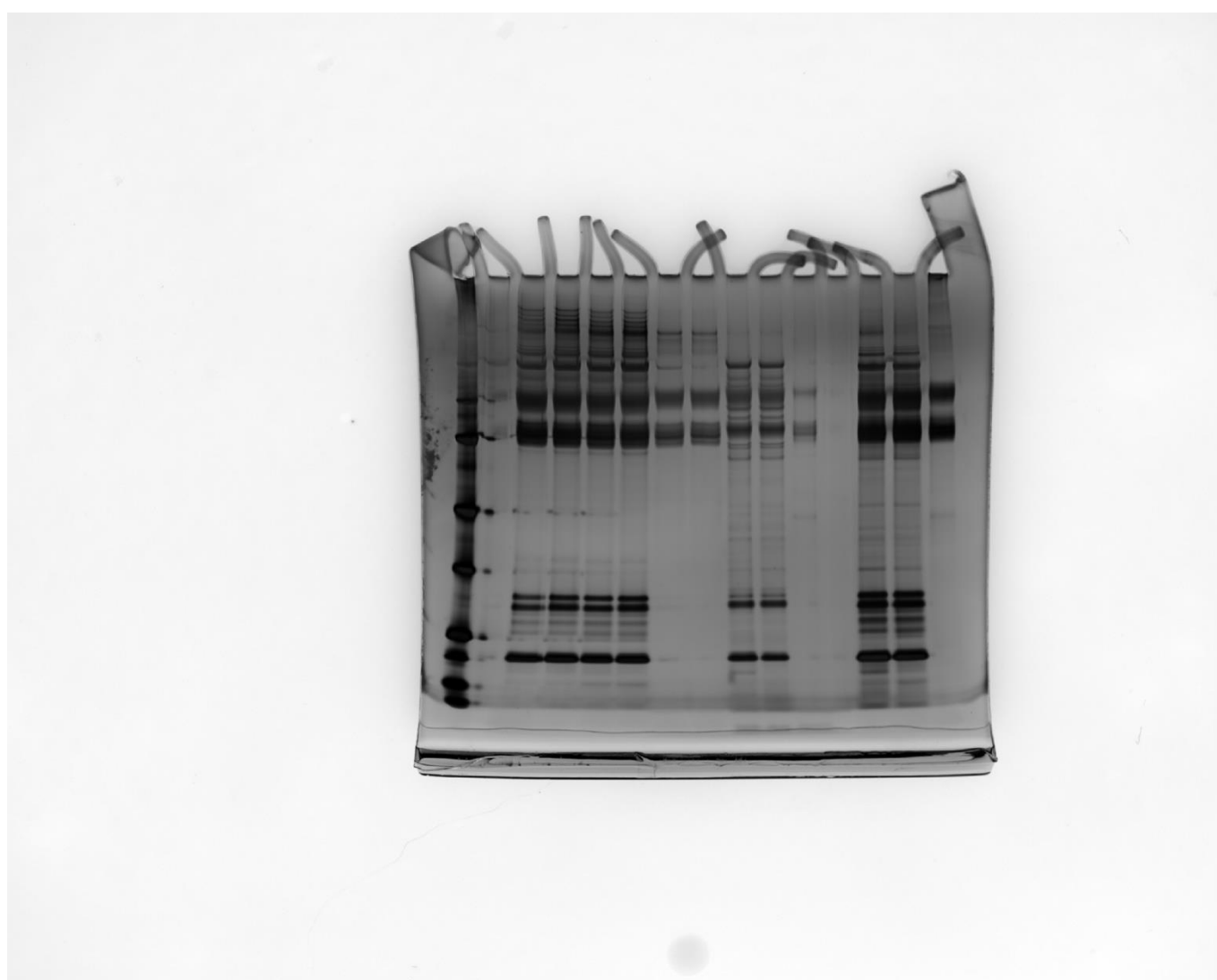

Flotation\_Sed5-COPII (Extended data Fig 2B)

Supplement: Supplementary file 5 — Unprocessed gels. [file 41594_2024_1413_MOESM5_ESM.pdf]
